# Zebrafish 3-*O*-Sulfotransferase-4 Generated Heparan Sulfate Mediates HSV-1 Entry and Spread

**DOI:** 10.1371/journal.pone.0087302

**Published:** 2014-02-03

**Authors:** Thessicar E. Antoine, Abraam Yakoub, Erika Maus, Deepak Shukla, Vaibhav Tiwari

**Affiliations:** 1 Departments of Ophthalmology and Visual Sciences & Microbiology/Immunology, University of Illinois at Chicago, Chicago, Illinois, United States of America; 2 Department of Microbiology and Immunology, Midwestern University, Downers Grove, Illinois, United States of America; Emory University School of Medicine, United States of America

## Abstract

Rare modification of heparan sulfate (HS) by glucosaminyl 3-*O* sulfotransferase (3-*O*ST) isforms generates an entry receptor for herpes simplex virus type-1 (HSV-1). In the zebrafish (ZF) model multiple 3-*O*ST isoforms are differentially expressed. One such isoform is 3-*O*ST-4 which is widely expressed in the central nervous system of ZF. In this report we characterize the role of ZF encoded 3-*O*ST-4 isoform for HSV-1 entry. Expression of ZF 3-*O*ST-4 into resistant Chinese hamster ovary (CHO-K1) cells promoted susceptibility to HSV-1 infection. This entry was 3-*O* sulfated HS (3-*O*S HS) dependent as pre-treatment of ZF 3-*O*ST-4 cells with enzyme HS lyases (heparinase II/III) significantly reduced HSV-1 entry. Interestingly, co-expression of ZF 3-*O*ST-4 along with ZF 3-*O*ST-2 which is also expressed in brain rendered cells more susceptible to HSV-1 than 3-*O*ST-4 alone. The role of ZF-3-*O*ST-4 in the spread of HSV-1 was also evaluated as CHO-K1 cells that expressed HSV-1 glycoproteins fused with ZF 3-*O*ST-4 expressing effector CHO-K1 cells. Finally, adding further evidence ZF 3-*O*ST-4 mediated HSV-1 entry was inhibited by anti-3*O* HS G2 peptide. Taken together our results demonstrate a role for ZF 3-*O*ST-4 in HSV-1 pathogenesis and support the use of ZF as a model to study it.

## Introduction

Heterogeneous chains of heparan sulfate (HS) are expressed on cell surface as a complex sulfated polysaccharide consisting of repeating disaccharide units of *N*-acetylglucosamine [GlcN] and glucuronic/iduoronic acid [GlcA/IdoA] [1]–[3]. The disaccharide chains in the HS are covalently linked to a serine residue of specific core protein via tetrasacchride link [GlcA-Gal-Gal-Xyl] making them a hybrid molecule with both protein and sugar component [3]. The chain of HS gets modified by series of multiple enzymes which includes glycosyltransferase, epimerase and sulfotransferases [4]. The first step of modification involves *N*-deactylation/*N*-sulfation of the glucosamine unit, followed by C5 epimerization of the glucuronic acid to iduronic acid, and *O*-sulfation of both residues. The last *O*-sulfation step is first catalyzed by 2-*O* sulfotransferase (2-*O*ST), followed by 6-*O* sulfotransferase (6-*O*ST) and finally 3-*O* sulfotransferases (3-*O*STs) enzyme, which are expressed in multiple isoforms [1]–[4]. It is well documented that the modification of HS is responsible for high sequence diversity or heterogeneity which in turn dictates functional specificity and versatility [3], [5], [6]. Therefore, specific 3-*O*ST isoforms potentially generate unique protein-binding sites within the HS chain which allows HSV-1 entry [5]–[10].

Interestingly, zebrafish (ZF) embryos are known to express multiple isoforms of heparan sulfate modifying enzyme 3-*O*-sulfotransferase (3-*O*ST) [11]–[13]. Cadwallader and Yost (2006b) characterized eight 3-*O*ST family members in ZF via *in situ* hybridization from early cleavage stage through 48 hr post fertilization with seven genes showing homology to known 3-*O*ST genes in mouse and humans [12]. The exclusive expression of 3-*O*ST-4 in central nervous system in ZF provided us a rationale to examine the role of 3-*O*ST-4 isoforms for a predominantly neurotropic virus HSV-1 [14]. Previously, we reported that enzymatic modifications in HS via 3-*O* sulfotransferases (3-*O*STs) isoforms other than ZF encoded 3-*O*ST-4 mediate HSV-1 entry and spread [15]–[18]. In this study our goal was to characterize ZF encoded 3-*O*ST-4 isoform for HSV-1 entry and spread. Our results here demonstrate that expression of ZF encoded 3-*O*ST-4 isoform in Chinese hamster ovary (CHO-K1) cells results in susceptibility to HSV-1 entry and spread. In addition we also demonstrate that our G2 peptide, which was isolated and characterized against 3-*O*ST-3 generated HS [19], [20], blocks HSV-1 entry into cells expressing ZF-3-*O*ST-4. The functional analogy between human and ZF 3-*O*ST-4 further validates the potential of ZF embryos for studying HSV infection.

## Results

### Chinese Hamster Ovary Cells (CHO-K1) Expressing Zebrafish (ZF) Encoded 3-*O*ST-4 Results in Susceptibility to HSV-1 Entry

**Figure 1:**
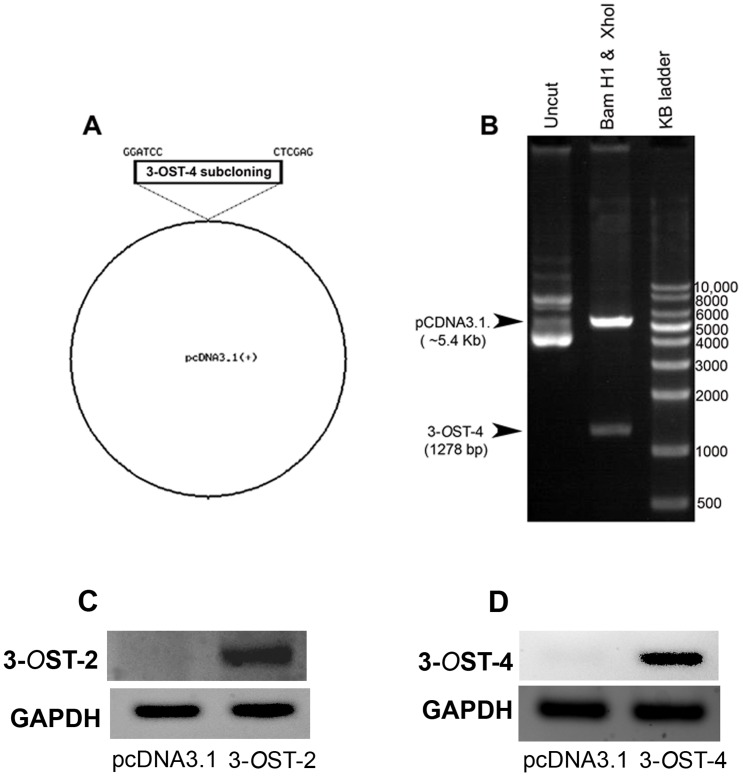
(A–B) Cloning of Zebrafish (ZF) encoded 3-*O*ST-4 isoform into pCDNA3.1. Zebrafish encoding 3-*O*ST-4 plasmid was constructed by inserting the open reading frame of 3-*O*ST-4 into pcDNA3.1 and the construct was designated pcDNA3.1-ZF-3-*O*ST-4. The inserted sequence of 1278 bp of 3-*O*ST-4 was verified after digestion using BamH1 and Xho1. (**C–D**). RT-PCR analysis for ZF encoded 3-*O*ST-2 and 3-*O*ST-4 isoforms expression in CHO-K1 cells. The housekeeping gene GAPDH was used as a normalization control.

The open reading frame of ZF encoding 3-*O*ST-4 gene was cloned into pcDNA3.1 for mammalian expression and the inserted sequence was verified by enzymatic digestion with restriction endonucleases (Fig. 1A and Fig. 1B). The expression of ZF encoded 3-*O*ST-2 [18] and 3-*O*ST-4 enzymes in Chinese hamster ovary (CHO-K1) cells was confirmed by RT-PCR analysis (Fig. 1C and Fig. 1D). In addition we also confirmed the transfection efficiency of ZF encoded both 3-*O*ST-2 and 3-*O*ST-4 in CHO-K1 cells by using GFP-expressing plasmid (Figure S1).

**Figure 2:**
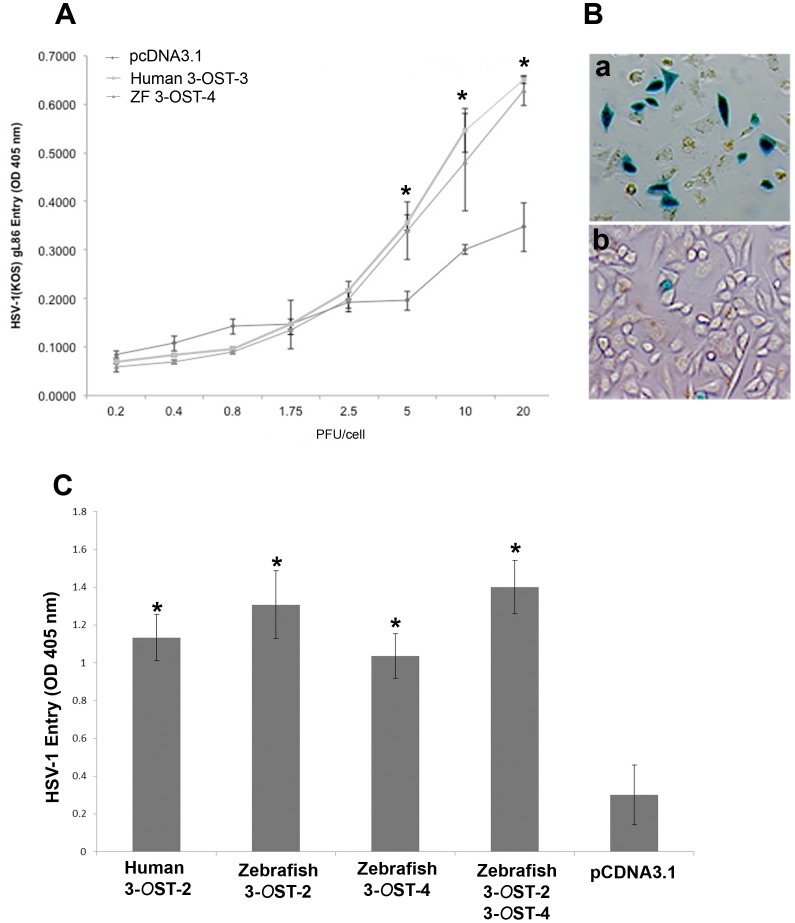
(A–C) Wild type Chinese hamster ovary (CHO-K1) cells expressing ZF 3-*O*ST-4 are susceptible to HSV-1 entry. (**A**). Dose response curve of HSV-1 entry into ZF expressing 3-*O*ST-4 CHO-K1 cells. Resistant wild-type CHO-K1 cells were transfected with ZF-3-*O*ST-4 at 2.5 µg DNA resulted HSV-1 gL86 entry, similar to human 3-*O*ST-3 expression. Cells transfected with empty vector pcDNA3.1 at 2.5 µg DNA was used as a negative control. Cultured cells were plated in 96-well plates and inoculated with two-fold serial dilutions of β-galactosidase-expressing recombinant virus HSV-1 (KOS) gL86 at the plaque forming units (PFU) indicated. After 6 hr, the cells were washed, permeabilized and incubated with ONPG substrate (3.0 mg/ml) for quantitation of β-galactosidase activity expressed from the input viral genome. The enzymatic activity was measured at an optical density of 410 nm (OD _410_). (**B**). HSV-1 entry into ZF 3-*O*ST-4 expressing CHO-K1 cells was further confirmed by X-gal staining. Cells grown (4×10^6^ cells) in six well dishes were challenged with β-galactosidase-expressing recombinant HSV-1 (gL86) at 20 pfu/cell. Wild-type CHO-K1 cells transfected with empty vector (pcDNA3.1) were also infected in parallel as negative control. After 6 h of infection at 37°C, cells were washed with PBS, fixed and permeabilized, and incubated with X-gal (5 bromo-4 chloro-3-indoyl- β-D- galactosidase) at 1.0 mg/ml, which yields an insoluble blue product upon hydrolysis by β-galactosidase. Blue cells (representing viral entry) were seen as shown. Microscopy was performed using a 20 × objective of Zeiss Axiovert 100. **C.** Co-expression of ZF encoded 3-*O*ST-2 and 3-*O*ST-4 resulted significant increase in HSV-1 infection. CHO-K1 cells cultured in 6 well dishes were transiently transfected with plasmids expressing human 3-*O*ST-2, ZF 3-*O*ST-2, ZF 3-*O*ST-4 and co-expressing ZF 3-*O*ST-2 and 3-*O*ST-4. CHO-K1 cells expressing pcDNA3.1 was used as negative control. Thirty six hr. post transfection cells were challenged with HSV-1 gL86 reporter virus. CHO-K1 cells expressing both 3-*O*ST-2 and 3-*O*ST-4 showed increase in HSV-1 entry. β-galactosidase based viral assay were performed using a soluble substrate o-nitrophenyl-β-D-galactopyranoside (ONPG; ImmunoPure, Pierce) using plate reader at 405 nm.

The ability of ZF encoded 3-*O*ST-4 was determined by transiently transfecting CHO-K1cells with ZF-3-*O*ST-4 expression plasmid and control pcDNA3.1 plasmid as a control. HSV-1 entry assay were performed by using reporter HSV-1 virus (HSV-1 gL86). As shown in Fig. 2 the dose response curve indicated HSV-1 entry into CHO-K1 cells expressing ZF 3-*O*ST-4 which was similar to human 3-*O*ST-3 cells while no HSV-1 entry was observed in CHO-K1 cells expressing empty vector pcDNA3.1 The results from ONPG was further confirmed by X-gal assay. As shown in Fig. 2B, X-gal (5-bromo-4-chloro-3-indolyl-β-D-galactosidase) staining was found to be positive for ZF-3-*O*ST-4 cells (Fig. 2B; panel a), the wild-type CHO-K1 cells expressing pcDNA3.1 empty vector remained resistant to HSV-1 entry (Fig. 2B; panel b).

### Co-expression of ZF 3-*O*ST-2 and 3-*O*ST-4 Enhances HSV-1 Entry

We next evaluated the effect of co-expression of ZF encoded 3-*O*ST-2 and 4 isoforms in the same cells. We reasoned to test this because both 3-*O*ST-2 and 3-*O*ST-4 are widely expressed in central nervous system of ZF [12]. CHO-K1 cells were co-transfected with plasmids expressing ZF 3-*O*ST-2 and 3-*O*ST-4. In parallel, CHO-K1 cells were individually transfected with 3-*O*ST-2 and 3-*O*ST-4 isoforms separately, CHO-K1 cells expressing human 3-*O*ST-2 and pcDNA3.1 was used as positive and negative control respectively. As indicated in Fig. 2C co-expression resulted in higher OD readings indicating the presence of both enzymes generate multiple sites for HSV-1 glycoprotein D (gD) and hence more viral entry.

### Heparinase Treatment to ZF 3-*O*ST-4 Cells Reduces HSV-1 Attachment and Entry

**Figure 3:**
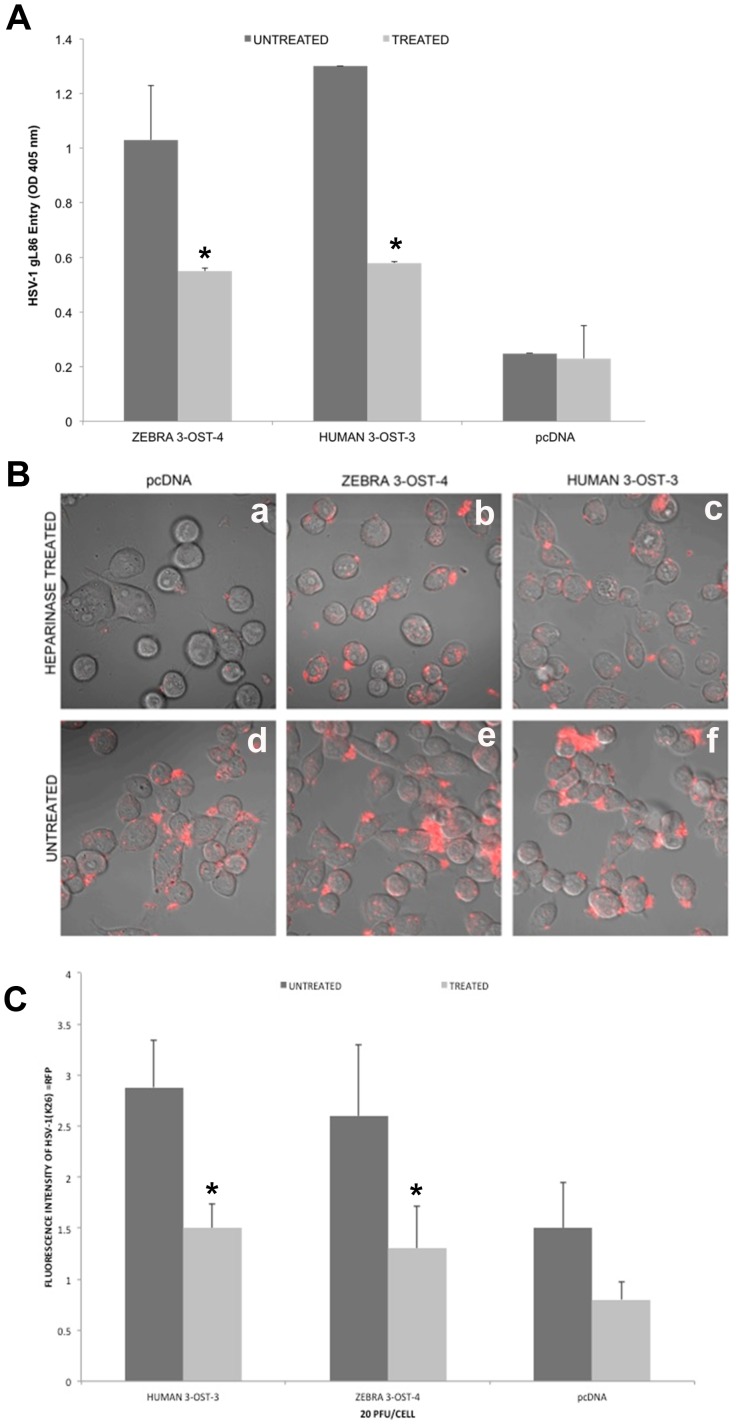
Enzymatic removal of cell surface heparan sulfate (HS) by heparinase treatment in ZF 3-*O*ST-4 expressing CHO-K1 cells reduces HSV-1 infection. Three groups of cultured CHO-K1 cells expressing empty vector pcDNA3.1 or ZF 3-*O*ST-4 or human 3-*O*ST-3 were treated with heparinase II/III (1.5 U/ml; grey bar) or mock treated (black bar) followed by exposing cells to HSV-1 (KOS) gL86 at 20 PFU/cell and viral entry was quantitated 6 hr later by ONPG assay (**panel A**) and fluorescent microscopy and quantification (panel B, and panel C). In the latter case HSV-1 capsid-tagged to RFP (HSV-1K26RFP) virus was used. The heparinase treated ZF-3-*O*ST-4 and human 3-*O*ST-3 cells had significantly lesser number of viral entry (**panel A**) compared to mock treated ZF-3-*O*ST-4 or 3-*O*ST-3 cells. Similarly confocal visualization (**panel B**) and quantification (**panel C**) resulted less red punctate of HSV-1K26RFP on heparinase treated ZF 3-*O*ST-4 or human 3-*O*ST-3 cells compared to mock treated cells.

Next, we evaluated the effect of enzymatic removal of 3-*O*S HS generated by ZF encoded 3-*O*ST-4 on HSV-1 entry by treating cells with a mixture of heparinase-II and -III (1.5 U ml/L). These enzymes selectively degrade HS chains by cleaving them [21]. For this experiment, CHO-K1 cells expressing ZF 3-*O*ST-4 along with CHO-K1 cells expressing human 3-*O*ST-3 were mock-treated or pretreated with heparinase-II/III before infecting with reporter β-galactosidase expressing HSV-1 gL86 [10]. As a negative control, CHO-K1 cells expressing empty vector pcDNA3.1 was treated with heparinase. As indicated in Fig 3A heparinase treated cells showed significant reduction in HSV-1 entry (light grey bar) compared to mock treated cells (dark grey bar). The above results were further confirmed by visual observation by using fluorescent HSV-1 K26RFP virus. Again the heparinase treated CHO-K1 cells expressing ZF 3-*O*ST-4 and human 3-*O*ST-3 showed significantly reduced number of cells binding/attaching with HSV-1K26RFP and therefore had reduced fluorescent intensity (Fig. 3B, upper panel) compared to mock treated ZF-3-*O*ST-4 and human 3-*O*ST-3 expressing cells (Fig. 3B, lower panel). The mock treated CHO-K1 cells expressing empty vector pcDNA3.1 did show HSV-1 attachment because of the presence of cell surface heparan sulfate, however heparinase treatment significantly affected HSV-1 binding and attachment. Similarly, the heparinase treatment resulted in decreased overall fluorescent intensity (light grey bar) suggesting the loss of virus binding compared to mock treated cells (dark grey bar) (Fig. 3C). The results indicate that heparinase treatment degrades HS including 3-*O*S HS on the cell surface thereby inhibiting HSV-1 entry in 3-*O*ST-4 cells similar to 3-*O*ST-3 cells.

### HSV-1 Glycoprotein Expressing Effector Cell Mediates Fusion with ZF Encoded 3-*O*ST-4 Expressing Target Cell

**Figure 4:**
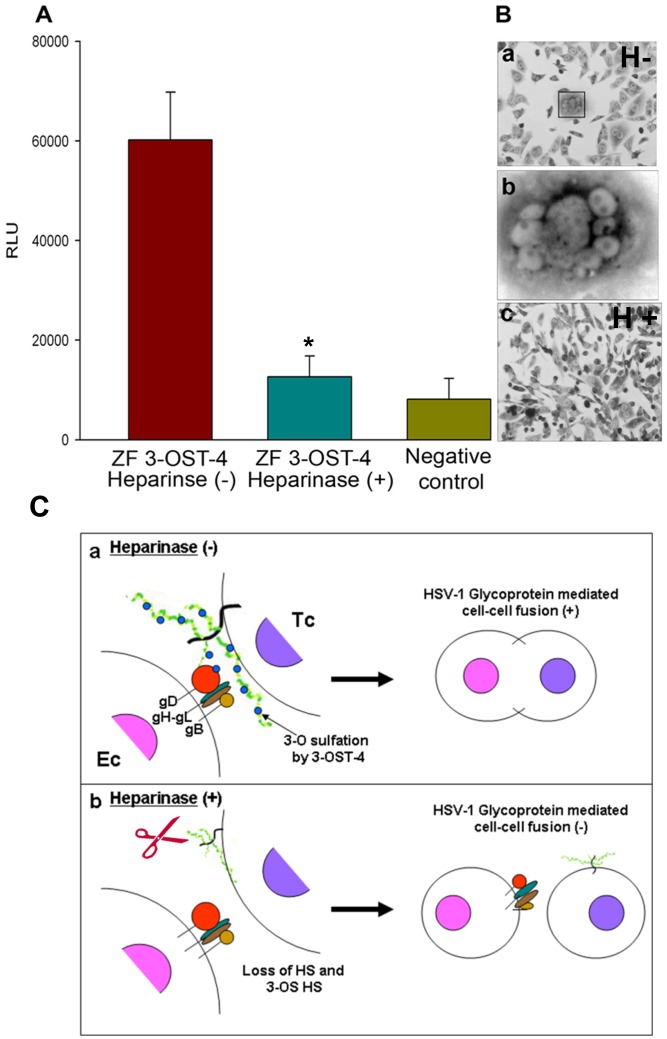
Effect of heparinase enzyme on Zebrafish (ZF) encoded 3-*O*ST-4 mediated cell to cell fusion with HSV-1 glycoprotein expressing cells (A–C). **A.** ZF 3-*O*ST-4-expressing target CHO-K1 cells gain the ability to fuse with effector cells co-expressing HSV-1 glycoproteins gB, gD, gH, and gL while heparinase treatment significantly blocks ZF 3-*O*ST-4 mediated fusion. The target CHO-K1 cells were transfected with plasmids expressing ZF 3-*O*ST-4 and luciferase reporter gene. The effector CHO-K1 cells were transfected with HSV-1 glycoproteins gB, gD, gH, and gL, and T7 RNA polymerase. CHO-K1 effector cells expressing control plasmid without HSV-1 glycoproteins were used as a negative control. In addition, target CHO-K1 cells expressing ZF 3-*O*ST-4 were treated with heparinase II/III (1.5 U/ml) (blur bar) or left untreated (red bar) for 1 hr prior to co-cultivation with effector CHO-K1 cells expressing four HSV-1 essential glycoproteins (gB, gD, gH-gL; 0.5 µg DNA each glycoprotein). A luciferase reporter assay was performed 24 h after the two cell populations were mixed together. Cell fusion was measured in relative luciferase units (RLUs) using a Sirius luminometer (Berthold Detection System). Similarly visual observation resulted multinucleated giant cells (**panel B**; subpanels a and b) with CHO-K1 cells expressing ZF 3-*O*ST-4 mixed with effector cells expressing HSV-1 glycoproteins. However hepainase treatment to ZF 3-*O*ST-4 cells resulted significant decrease in giant cell formation. **Panel c** represent cartoon indicating cell fusion in mock treated ZF 3-*O*ST-4 cells (subpanel a) vs. heparinase treated ZF 3-*O*ST-4 cells inhibiting HSV-1 glycoprotein mediated cell fusion (subpanel b).

We next examined the role of ZF 3-*O*ST-4 in HSV-1 spread by using HSV-1 glycoprotein mediated cell-to-cell fusion assay. We purposefully used wild type CHO-K1 cells because they lack endogenous glycoprotein D (gD) receptor required for HSV-1 entry. [10]. To quantify the HSV-1 glycoprotein induced cell fusion between 3-*O*S HS cells modified by 3-*O*ST-4 and HSV-1 glycoproteins a luciferase reporter gene assay was performed [22], [23]. Wild type CHO-K1 cells were transiently transfected with each of four glycoprotein plasmids: pPEP98 (gB), pPEP99 (gD) pPEP100 (gH), and pPEP101 (gL), as well as, the plasmid pT7EMCLuc that expresses a luciferase reporter gene was considered “effector” cell. In parallel “target” cells were transfected with a 3-*O*ST plasmid expressing ZF encoded 3-*O*ST-4 and the plasmid pCAGT7, which expresses T7 RNA polymerase to induce expression of the Luciferase gene. For a negative control, cells were transfected with T7 RNA polymerase and control plasmid pcDNA3.1. The cells expressing human 3-*O*ST-4 and T7RNA polymerase served as a positive control. As shown in Fig. 4 a high amount of fusion occurred in ZF encoded 3-*O*ST-4 expressing cells (red bars) compared to the negative control (yellow bar). Clearly, the 3-*O*S HS generated by ZF encoded 3-*O*ST-4 is capable of mediating cell fusion as well. These results reinforce our findings that CHO-K1 cells expressing ZF 3-*O*ST-4 allow cell fusion to occur, and thus potentially could facilitate spread of HSV-1 in a ZF model. We also evaluated if enzymatic removal of ZF 3-*O*ST-4 cells affects HSV-1 glycoprotein mediated cell fusion. In this experiment, CHO-K1 cells expressing ZF 3-*O*ST-4 were treated separately with heparinase I and heparinase II (1.5 U ml/L) or mock treated with 1 × PBS for 45 minutes before mixing cells with effector cells expressing HSV-1 glycoproteins; gB, gD, gH-gL. The two population, effector and target cells were mixed in equal 1∶1 ratio and co-cultivated. As shown in Fig 4 cells ZF 3-*O*ST-4 treated with heparinase-II/III showed significant reduction in fusion (Fig. 4; blue bars) as well as reduced polykaryocytes formation (Fig. 4B, panel c), while mock treated cells forms multinucleated giant cells or polykaryocytes (Fig. 4B, panels a and b). Cartoon in Fig. 4C panel a, demonstrates that mock treated ZF 3-*O*ST-4 cells were able to fuse with effector cells, while heparinase treatment to ZF3-*O*ST-4 cells resulted no fusion (Fig. 4C, panel b).

### Phage Display Library Screening Derived Anti-3-*O*S HS (G2) Peptide Blocks HSV-1 Entry in ZF 3-*O*ST-4 Cells

**Figure 5:**
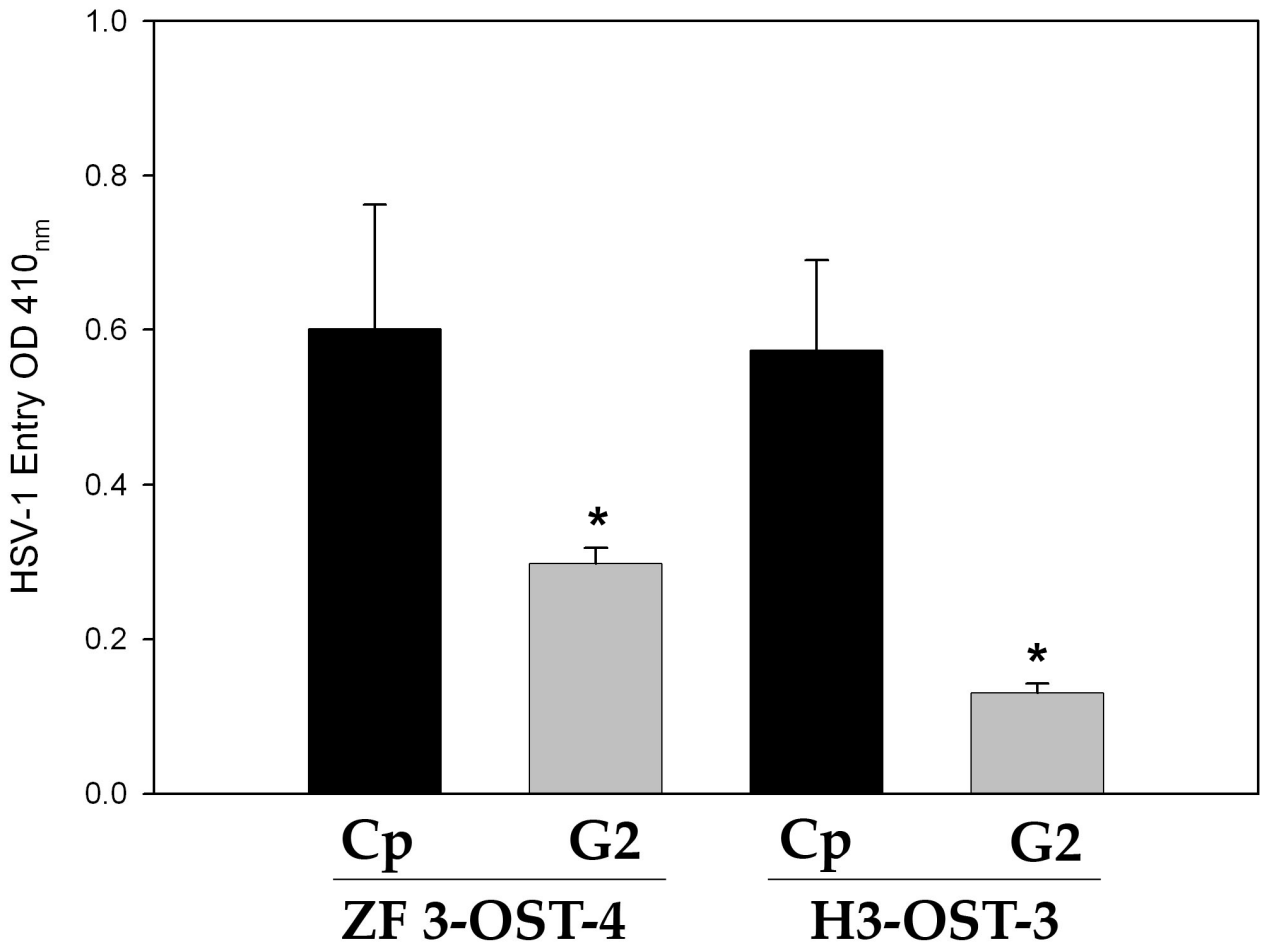
Anti-3OS HS (G2) peptide block HSV-1 entry into ZF 3-*O*ST-4 cells. CHO-K1 cells expressing ZF 3-*O*ST-4 and human 3-*O*ST-3 were pretreated for 60 min with 0.2 mM concentrations of G2, or control peptide (Cp) peptides. Pretreated cells were infected with a β galactosidase-expressing recombinant virus HSV-1(KOS) gL86 (20 pfu/cell) for 6 h. Viral entry was measured via microplate reader at 405 nm.

We finally tested 12-mer G2 peptide that was isolated and characterized for binding to human 3-*O*ST-3 generated heparan sulfate (HS) and blocking its role in viral entry [19], [20]. The CHO-K1 cells expressing ZF 3-*O*ST-4 were pre-treated with G2 peptide or a control peptide (Cp) before infecting them with HSV-1 reporter virus. CHO-K1 cells expressing human 3-*O*ST-3 treated with G2 peptide was used as a control. As indicated in the Fig. 5, the pre-treatment of ZF-3-*O*ST-4 expressing cells resulted in loss of HSV-1 entry.

## Materials and Methods

### Plasmids

Zebrafish (ZF) encoded 3-*O*ST-4 gene was cloned into pcDNA3.1 plasmid (Genscript). Human 3-*O*ST-3 expressing plasmid (pDS43) was generous gifts from Dr. Deepak Shukla (University of Illinois at Chicago) [10]. The plasmid used in the cell fusion study includes HSV-1 (KOS) glycoprotein expressing plasmids used were pPEP98 (gB), pPEP99 (gD), pPEP100 (gH), and pPEP101 (gL) [22], along with pCAGT7 (T7 RNA polymerase), pT7EMCLuc (luciferase gene) for the luciferase assay, and a control empty vector pCDNA3.1 from Invitrogen (Carlsbad, CA, USA).

### RT-PCR for ZF Encoded 3-*O*ST-2 and 3-*O*ST-4 Enzyme Expressions

CHO-K1 cells were transfected with 2.5 ug of ZF encoded 3-*O*ST-2, and 3-*O*ST-4, or with empty vector (pcDNA3.1) plasmid using lipofectamine2000 (Invitrogen) according to the manufacturer’s recommendations. 24 hrs, post-transfection, cells were washed in PBS, and RNA was extracted using TRIzol (Invitrogen)-chloroform extraction method as per the manufacturer’s protocols. cDNA synthesis was performed using High Capacity cDNA Reverse Transcription Kit (Applied Biosystems). PCR for each gene was performed using PCR Master Mix (Fermentas) and the following primer sets: ZF 3-*O*ST-2 Forward 5′-TGTCCCAGATCCACTTTGTG-3′ and Reverse 5′- AAGTAGAAGTGCTTGTCCGTG-3′, amplifying a product size of 118 bp, ZF 3-*O*ST-4 Forward 5′-CATGTTCACCCTCTCCCTTTC-3′ and Reverse 5′- AGTAAAGTTTCTCCCTCAGCG-3′, amplifying a product size of 149 bp. The housekeeping gene GAPDH, used as a normalization control, was amplified using the following primers: Forward 5′- TTCACCACCATG GAGAAGG-3′ and Reverse 5′- AGAAGGGGCGGAGATGAT-3′, product size 72 bp. PCR products were electrophoresed on a 2% agarose-ethidium bromide gel, and then bands were visualized using Image Quant LAS4000 digital image system (GE Life Sciences).

### Cell Culture and Viruses

CHO-K1 cells were grown in Ham’s F-12 medium (Gibco/BRL, Carlsbad, CA, USA) supplemented with 10% fetal bovine serum (FBS), and penicillin and streptomycin (Gibco/BRL). Wild-type Chinese hamster ovary-K1 (CHOK1) cells and β-galactosidase expressing recombinant HSV-1 (KOS) gL86 were kindly provided by P.G. Spear (Northwestern University, Chicago).

### Validation of ZF 3-*O*ST-2 and 3-*O*ST-4 Transfection Efficiency by Cotransfection with pEGFP-N1

The transfection efficiency of ZF encoded 3-*O*ST-2 and 3-*O*ST-4 was verified by co-transfection with pEGFP-N1. CHO-K1 cells were transfected with pcDNA3.1, ZF 3-*O*ST-2, or ZF 3-*O*ST4 and pEGFP-N1 (Clontech) plasmid using lipofectamine2000. 24 hrs, post-transfection, cells were washed in PBS, and imaged using Zeiss Axiovert100 Inverted microscope (Zeiss), and image analysis was performed using MetaMorph software.

### HSV-1 Entry Assays

Chinese hamster ovary (CHO-K1) cells grown in a 6-well plates were transfected with 2.5 µg of human or ZF encoded 3-*O*ST isoforms (3-*O*ST-2,or 4), or pCDNA3.1 using LipofectAMINE (Gibco/BRL). At 16 h posttransfection, the cells were replated into 96-well dishes for infection with recombinant virus. After 6-h post infection, β-galactosidase assay were performed using either a soluble substrate o-nitrophenyl-β-D-galactopyranoside (ONPG; ImmunoPure, Pierce) or X-gal (Sigma). For the soluble substrate, the enzymatic activity was measured at 410 nm using a micro-plate reader. For X-gal assay, the cells were fixed (2% formaldehyde and 0.2% glutaraldehyde) and permeabilized (2 mM MgCl2, 0.01% deoxycholate, and 0.02% nonidet NP-40 (Sigma). Finally, 1 mL of β-galactosidase reagent (1.0 mg/ml X-gal in ferricyanide buffer) was added to each well and incubated at 37°C for 90 min before the cells were examined using bright field microscopy under the 20 × objective (Zeiss, Axiovert 100M). Heparinase-II and -III were obtained from Sigma–Aldrich Chemical.

### Cell Fusion Assays

A cell-to-cell fusion assay described previously was used [23]. CHO-K1 cells were grown in 6-well plates to subconfluent levels. The so-called “target” cells were transfected with ZF encoded plasmids expressing 3-*O*ST-4 and the luciferase gene. The “effector” or virus-like cells were co-transfected with plasmids expressing HSV-1 glycoproteins gB, gD, gH, and gL, and T7 RNA polymerase. In either case, the total amount of DNA used for transfection was kept constant. After 16 h, target and effector cells were mixed in a 1∶1 ratio and then replated in 24-well dishes. The activation of the reporter luciferase gene as a measure of cell fusion was examined after 24 h. To demonstrate sensitivity to heparinase treatment target CHO-K1 cells expressing ZF 3-*O*ST-4 were treated with a 1∶1 mixture of heparinase-II/III for 2 h prior to mixing with the effector cells. Target cells were mock treated with the buffer alone to serve as a control.

### Confocal Imaging

CHO-K1 cells were cultured in 35 mm glass-bottom dishes (MatTek Corporation) for 24 h. The following day cells were infected with HSV-1(K26) RFP virus for 2 h at 4°C. At the end of the incubation cells were washed with 1x PBS. Images were captured at 63x/1.40 Oil objective using Zeiss Confocal 710 (Jena, Germany). The fluorescence intensity was analyzed with Zen 2011 software.

## Conclusions

ZF model expresses wide range of heparan sulfate modifying 3-*O* sulfotransferases (3-*O*ST) enzymes including 3-*O*ST-4 isoform during physiological development [11]–[13], [24]. Interestingly both 3-*O*ST-2 and 3-*O*ST-4 isoforms are predominantly expressed in different regions of ZF brain and there is a possibility that HSV-1 being neurotropic virus may exploit one or both these isoforms during neuronal invasion in ZF model. Our previous study has already indicated the role ZF 3-*O*ST-2 isoform for HSV-1 entry [18]. In this study we characterized 3-*O*ST-4 isoform for HSV-1 entry and spread.

**Figure 6:**
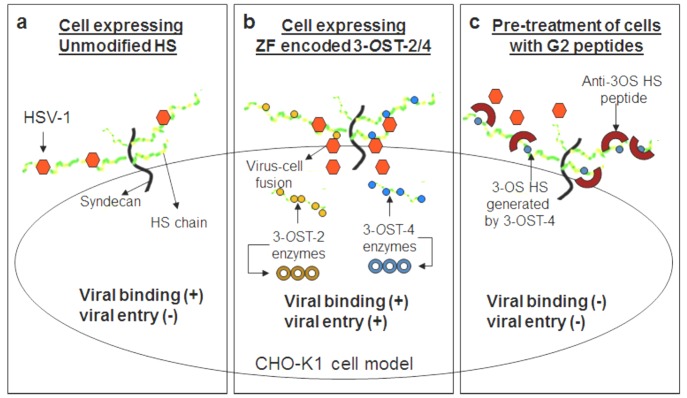
Proposed model for ZF 3-*O*ST-4 mediated HSV-1 entry. Cell expressing unmodified heparan sulfate (HS) do allow viral binding but not viral entry (panel a), while cells expressing HS modifying enzymes 3-*O* sulfotransferases (3-*O*ST) such as ZF encoded 3-*O*ST-2 or 3-*O*ST-4 mediates both viral binding and entry (panel b). Pre-treatment of CHO-K1 cells expressing ZF encoded 3-*O*ST-4 with anti-3-*O*S HS (G2) peptide generated against human 3-*O*ST-3 binds to the sites on the modified HS used by HSV-1 and thereby prevents both viral binding as well as viral entry (panel c).

At the beginning of our study ZF encoding 3-*O*ST-4 region was successfully cloned into empty vector pcDNA3.1. The resultant construct allowed us to successfully express 3-*O*ST-4 in resistant CHO-K1 cells (Fig. 1D), which became susceptible to HSV-1 entry upon ZF 3-*O*ST-4 expression (Fig. 2). In addition, CHO-K1 cells expressing 3-*O*ST-4 allowed cell-to-cell fusion as an indicator of HSV-1 spread. Both the events of HSV-1 entry and spread were HS dependent as evident from enzymatic treatment of cells which resulted in significant decrease in HSV-1 infection (Fig. 4). Further, we provided evidence that by blocking ZF modified HS by using phage display derived anti-3-*O*S HS (G2) peptide HSV-1 entry was significantly reduced. Interestingly co-expression of both 3-*O*ST-2 and 3-*O*ST-4 resulted in higher viral entry. ZF 3-*O*ST-2 and 3-*O*ST-4 are highly expressed in forebrain, hindbrain, and olfactory epithelium of central nervous system [12]. Taken together our findings strongly suggest that ZF 3-*O*ST-4 mediates HSV-1 entry and cell-fusion similar to human 3-*O*ST-3 and 3-OST-4 isoforms. It is also very interesting that HSV-1 entry was enhanced with the co-expression of 3-*O*ST-2 and -4 isoforms, which means their co-expression naturally in ZF brain can result in higher infection. We also provide additional new information that virus entry via ZF 3-*O*ST-4 isoform was inhibited by anti-3-*O*S HS peptide (Fig. 5) as suggested in proposed model (Fig. 6). Our results not only extend the list of 3-*O*STs receptors for HSV-1 entry into ZF model [24] but also provide new information related to the mechanism needed to infect brain tissues of ZF. While ZF 3-*O*ST-2 is widely expressed in CNS, ZF-3-*O*ST-4 is expressed in various regions of CNS and also in the eye tissues [12]. Similar cell and tissue specific 3-*O*ST-2 and 3-*O*ST-4 expression profiles for human and mouse model has been suggested [25], [26]. Therefore, ZF embryo model to study HSV-1 infection could be very useful for various reasons. For instance, fine alterations of GAG modification is a dynamic event during zebrafish development which is regulated as the ZF embryos age [27]. ZF embryos to adult form might show variability in susceptibility to HSV-1 infection, which in turn could shed new light on how the modifications within HS play a role in viral infectivity. Similarly, HSV-1 tropism in ZF embryo may very well be guided by 3-*O*STs expression especially in the brain or in the eye tissues. In this regard our phage display generated anti-3-*O*S HS (G2) peptide [19] will be useful to study its ability to affect HSV-1 entry and spread in live ZF embryos. Alternatively, a micro-injection based strategy using anti-3-*O*S HS peptides fused with cargo-nanoparticles will be useful to enhance efficacy of anti-HSV-1 activity in targeted specific tissue rich in HS sulfation. Overall, our data confirms the role of ZF 3-*O*ST-4 in HSV-1 infection and supports the use of ZF model to study HSV-1 infection.

## Supporting Information

Figure S1 **The transfection efficiency of both zebrafish encoded 3-** ***O*** **ST isoforms (3-** ***O*** **ST-2 and 3-** ***O*** **ST-4) was verified via co-transfection with GFP (pGFP-N1) expressing plasmid (panel a: bright field; panel b: GFP expression and panel c: overlay).** Zeiss Axiovert 100 inverted microscope was used for imaging.
(JPG)
