# Supplementary figures and images for: Zebrafish 3-O-Sulfotransferase-4 Generated Heparan Sulfate Mediates HSV-1 Entry and Spread
Source: PLoS One. 2014 Feb 3;9(2):e87302. doi: 10.1371/journal.pone.0087302 (PMC3911948; doi:10.1371/journal.pone.0087302)

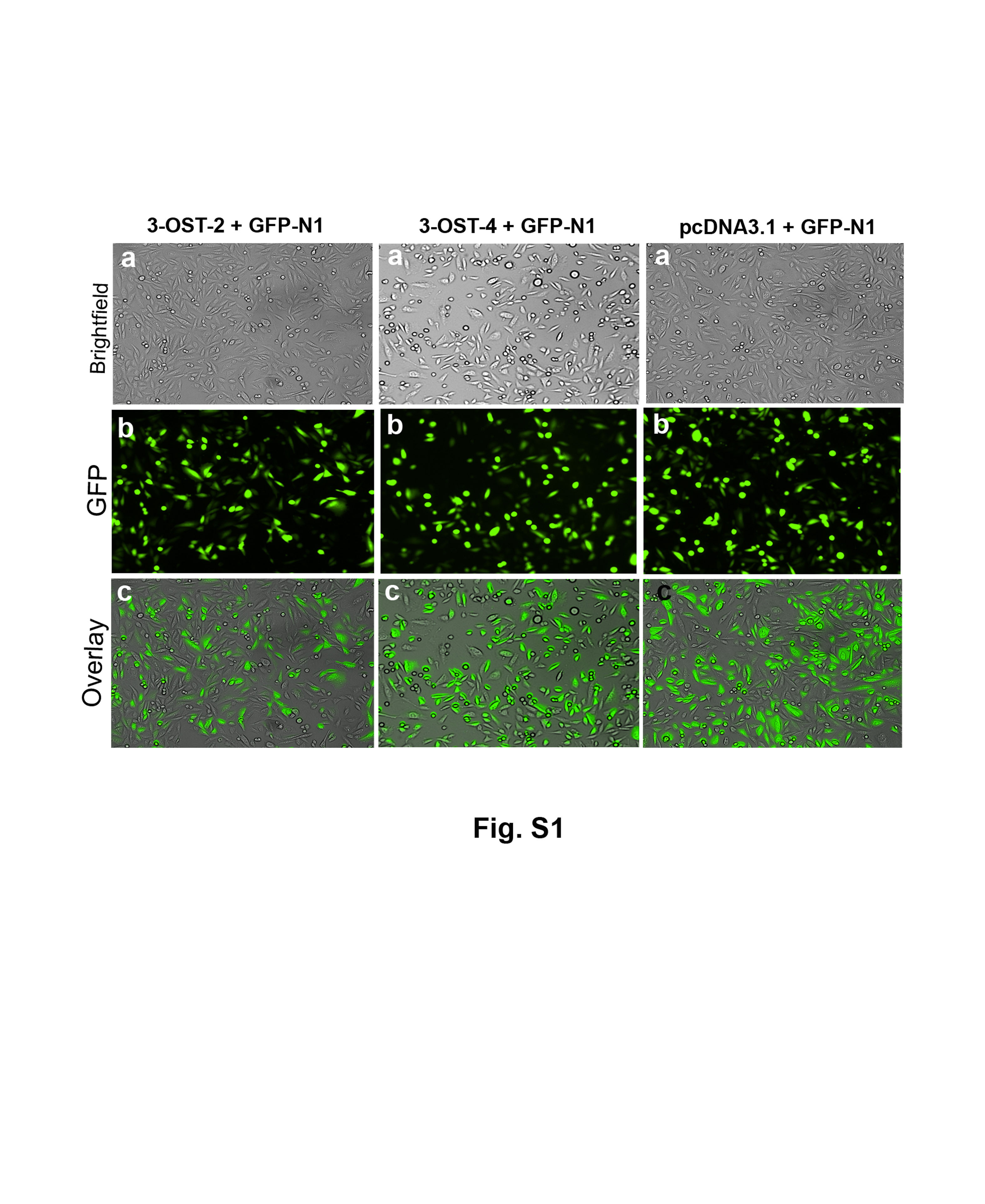

Supplement: Figure S1 — The transfection efficiency of both zebrafish encoded 3- O ST isoforms (3- O ST-2 and 3- O ST-4) was verified via co-transfection with GFP (pGFP-N1) expressing plasmid (panel a: bright field; panel b: GFP expression and panel c: overlay). Zeiss Axiovert 100 inverted microscope was used for imaging. (JPG) [file pone.0087302.s001.jpg]
